# Bio-Approach for Obtaining Enantiomerically Pure Clopidogrel with the Use of Ionic Liquids

**DOI:** 10.3390/ijms241311124

**Published:** 2023-07-05

**Authors:** Joanna Chałupka, Adam Sikora, Marta Ziegler-Borowska, Michał Piotr Marszałł

**Affiliations:** 1 Department of Medicinal Chemistry, Faculty of Pharmacy, Collegium Medicum in Bydgoszcz, Nicolaus Copernicus University in Toruń, Dr. A. Jurasza 2, 85-089 Bydgoszcz, Poland; joanna.chalupka@cm.umk.pl (J.C.); mmars@cm.umk.pl (M.P.M.); 2 Department of Biomedical Chemistry and Polymer Science, Faculty of Chemistry, Nicolaus Copernicus University in Torun, Gagarina 7, 87-100 Toruń, Poland; martaz@umk.pl

**Keywords:** clopidogrel, ionic liquid, enantioselective biotransformation, lipase, *Candida rugosa*

## Abstract

Clopidogrel is a chiral compound widely used as an antiplatelet medication that lowers the risk of blood clots, strokes, and heart attacks. The main aim of the study presented herein was to obtain (*S*)-clopidogrel, which is commercially available in treatments, via the kinetic resolution of racemic clopidogrel carboxylic acid with the use of lipase from *Candida rugosa* and a two-phase reaction medium containing an ionic liquid. For this purpose, the enantioselective biotransformation of clopidogrel carboxylic acid and chiral chromatographic separation with the use of a UPLC-MS/MS system were optimized. The best kinetic resolution parameters were obtained by using a catalytic system containing lipase from *Candida rugosa* OF as a biocatalyst, cyclohexane and [EMIM][BF4] as a two-phase reaction medium, and methanol as an acyl acceptor. The enantiomeric excess of the product was ee_p_ = 94.21% ± 1.07 and the conversion was c = 49.60% ± 0.57%, whereas the enantioselectivity was E = 113.40 ± 1.29. The performed study proved the possibility of obtaining (*S*)-clopidogrel with the use of lipase as a biocatalyst and a two-phase reaction medium containing an ionic liquid, which is in parallel with green chemistry methodology and does not require environmentally harmful conditions.

## 1. Introduction

Cardiovascular disease (CVD), including transient ischemic attack (TIA), acute coronary syndrome (ACS), and peripheral artery disease (PAD) and minor strokes, are primarily treated and secondarily prevented with antiplatelet therapy. The P2Y12 inhibitors, of which clopidogrel was one of the first, are crucial components of antiplatelet therapy, which helps treat and prevent secondary cardiovascular disease (CVD). Clopidogrel is frequently used in conjunction with aspirin as a part of dual antiplatelet treatment (DAPT) for the secondary prevention of ACS. In randomized studies of ACS patients, newer and more effective P2Y12 inhibitors (ticagrelor and prasugrel) have demonstrated a more significant reduction in ischemia risk than clopidogrel. However, these more recent, more potent P2Y12 inhibitors still have limitations [1,2,3,4,5,6,7,8,9,10]. Therefore, clopidogrel is still one of the most frequently used antiplatelet medicines. 

Clopidogrel is a thienopyridine derivative prodrug that, in order to exert its antithrombotic effect, must first be activated by the liver. The active metabolite selectively inhibits adenosine diphosphate-induced platelet aggregation by irreversibly inhibiting the platelet adenosine diphosphate P2Y12 receptor [3,11,12,13,14]. Clopidogrel is an enantiomeric medication that undergoes metabolic conversion to activate its (*S*)-configuration. When administered in high dosages in animals, the (*R*)-enantiomer of the activated form of clopidogrel has the potential to cause convulsions and is devoid of any antithrombotic efficacy [15]. As a result, only the (*S*)-enantiomer of clopidogrel is commercially available in treatment [16]. 

According to the European Society of Cardiology (ESC) guidelines on DAPT (2017), for acute myocardial infarctions in patients presenting with an ST-segment elevation myocardial infarction (STEMI) (2017), revascularization (2018), the diagnosis and management of chronic coronary syndromes (2019), and acute coronary syndromes in patients presenting without a persistent ST-segment elevation (NSTE-ACS) (2020), clopidogrel is one of the P2Y12 receptor inhibitors associated with an excellent safety profile, mainly in terms of gastrointestinal and skin disorders, allergies, and neutropenia. The wide variability in the pharmacodynamic response to clopidogrel is connected to various factors, including genotype polymorphisms. In patients with chronic coronary syndromes that are undergoing or have undergone percutaneous coronary interventions (PCI), clopidogrel is the default P2Y12 inhibitor in addition to acetylsalicylic acid (ASA). Similarly, suppose oral anticoagulation is needed (e.g., for stroke prevention in atrial fibrillation) in patients with chronic coronary syndrome. In that case, triple therapy should be initiated with an oral anticoagulant (OAC), ASA, and clopidogrel in patients with a moderate or high risk of stent thrombosis, irrespective of the type of stent used [17,18,19]. Considering the information mentioned above, clopidogrel remains the preferable medicine for treating cardiovascular disorders requiring P2Y12 inhibition. 

There are now three basic methods for acquiring optically pure substances, which is an asymmetric organic synthesis using pro-chiral substrates, racemate separation, and a “chiral pool” of optically pure substrates. A high selectivity, more favorable reaction conditions, and biocompatibility are just a few of the positive characteristics that make enzyme-mediated transformations so valued as a potent alternative instrument in organic synthesis. Since organic synthesis relies on performing a stereoselective biotransformation, and is therefore significantly less expensive than using a “chiral pool,” kinetic resolution (racemate resolution) with the use of enzymes is one of the most widely used techniques [20,21,22,23,24,25,26,27,28,29,30,31]. It also avoids the use of environmentally hazardous and toxic chemical compounds.

Organic solvents are still customary as reaction media in the stereoselective biotransformation of racemic forms of active pharmaceutical substances. However, most of these substances are poisonous and hazardous to the environment, and in many instances, they can contaminate the final synthesis product with organic molecules. On the other hand, ionic liquids offer several benefits when used as the reaction medium. Ionic liquids are frequently referred to as “design solvents” since they have been designed to address various synthetic issues. They are helpful in numerous technical processes due to their unique characteristics [32,33,34,35,36]. Ionic liquids are regarded as “green solvents” because they have a number of distinctive properties, such as an extremely low vapor pressure and a high thermal stability, which offer advantages such as the ease of containment, product recovery, and a recycling ability as well as a high ionic conductivity and a high solvation power.

## 2. Results and Discussion

### 2.1. Enantioselective Biotransformation of Racemic Clopidogrel

**Figure 1:**
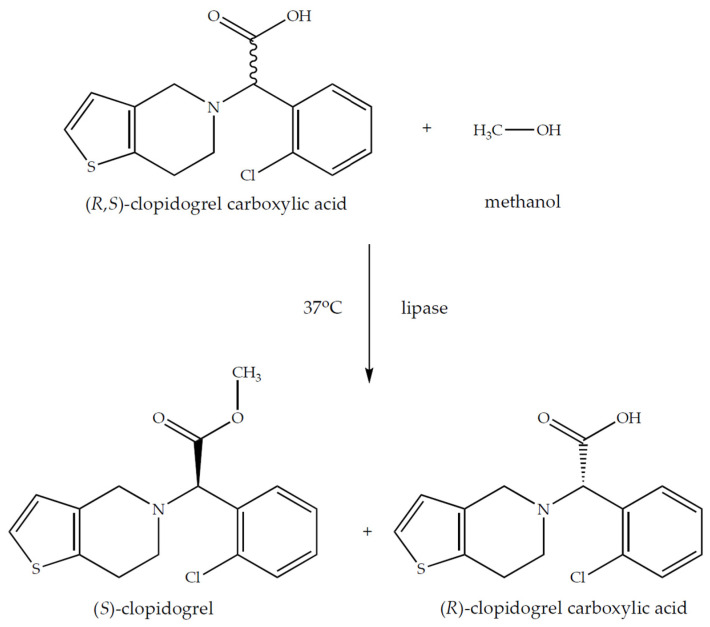
Enantioselective esterification of (*R*,*S*)-clopidogrel carboxylic acid with the use of *Candida rugosa* lipase as the biocatalyst. The reaction mixture consisted of (*R*,*S*)-clopidogrel carboxylic acid (3.5 mg, 0.01 mM); methanol (3.0 µL, 0.25 mM); lipase from *Candida rugosa* OF (10.0 mg); and *n*-hexane, *n*-heptane, or cyclohexane (5 mL) with or without the addition of an ionic liquid (100 µL), and was incubated for 5 days (120 h) along with shaking (250 RPM) at 37 °C.

The enantioselective biotransformation of (*R*,*S*)-clopidogrel carboxylic acid was examined under various conditions using commercially available lipase from *Candida rugosa* OF (Figure 1). The research investigated several reaction systems to avoid the solubility problem with the racemic molecule. The created and tested catalytic systems utilized several kinds of ionic liquids, such as: [EMIM][EtSO_4_], [EMIM][MSF_3_], [HMIM][BF_4_], [EMIM][BF_4_], and [DMIM][MeSO_4_]. Nevertheless, only a few of the tested reaction systems met the required performance standards for kinetic resolution (Table 1). In every instance, it was seen during the studies that the conversion value increased with the reaction time.

The reaction containing cyclohexane and [EMIM][BF4] as the rection medium produced the best results out of all the investigated catalytic systems. The (*S*)-clopidogrel was produced after 120 h of incubation, with the maximum value of enantioselectivity equaling E = 113.4 ± 1.20 and the product’s enantiomeric excesses equaling ee_p_ = 94.21% ± 1.07%. Although using other tested reaction systems enabled the achievement of passable results, the enantiomeric purity of the products was inferior.

### 2.2. Effect of Reaction Time

**Figure 2:**
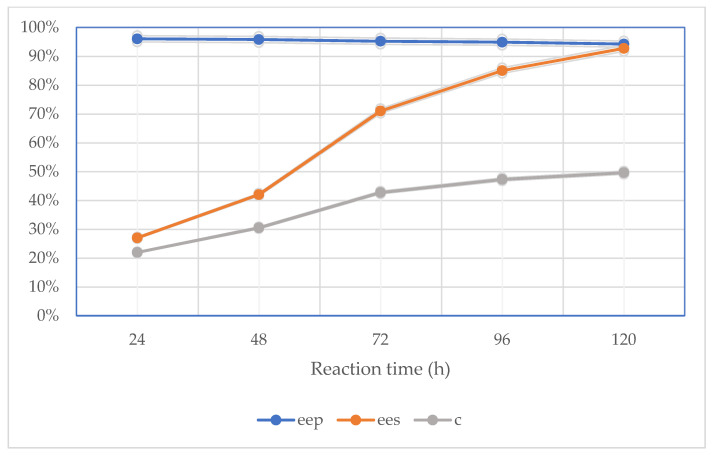
Effect of reaction time on the enzymatic parameters of performed kinetic resolution of (*R*,*S*)-clopidogrel carboxylic acid in the two-phase catalytic system consisting of [EMIM][BF_4_] and cyclohexane, including values of enantiomeric excesses of both substrates (*ee_s_*) and products (*ee_p_*) as well as conversion (*c*) and enantioselectivity (*E*).

**Figure 3:**
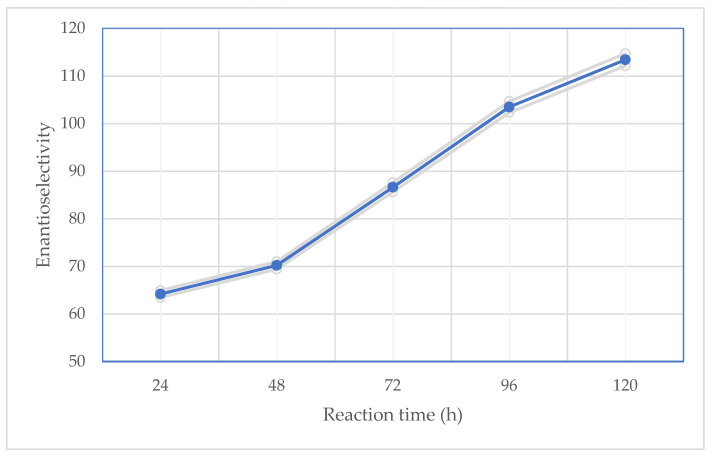
Effect of reaction time on the enzymatic parameters of performed kinetic resolution of (*R*,*S*)-clopidogrel carboxylic acid in the two-phase catalytic system consisting of [EMIM][BF_4_] and cyclohexane, including values of enantioselectivity (*E*).

One of the most affecting aspects of the kinetic resolution of racemic compounds was found to be the incubation duration of the reaction mixture among all the evaluated influencing factors on enzyme-catalyzed biotransformations. Other investigations have shown that the enantioselectivity and enantiomeric excess of both products and substrates rapidly decline when the reaction medium is incubated excessively. The lack of a substrate makes the reaction no longer regarded as enantioselective because the conversion value has the potential to be higher than 50%. Commercially available lipases from *Candida rugosa* OF, methanol (3 µL) as an acyl acceptor, (*R*,*S*)-clopidogrel carboxylic acid (3.0 mg), an ionic liquid (100 µL), and an organic solvent (5 mL) were utilized as the reaction media in the experiment. The biotransformations were carried out for 120 h at 37 °C. According to Figure 2 and Figure 3, the enantiomeric excess of the substrates, the conversion, and the enantiomeric ratio increased along with the reaction duration. Over the same period, the product’s enantiomeric excess slowly diminished. The conversion value was the highest after 120 h of reaction (Figure 2), and it varied depending on the type of catalytic system (Table 1).

### 2.3. Effect of Reaction Medium

The investigated catalytic systems were effective in the reaction media both with and without ionic liquids. Nevertheless, it was observed that *Candida rugosa* lipase OF exhibited various catalytical properties depending on the type of reaction system. Due to this, one of the most crucial aspects of improving reaction conditions to increase enantioselectivity is selecting the best reaction medium. Therefore, the effect of five different ILs was tested for the production of (*S*)-clopidogrel. Table 1 shows that the type of utilized ionic liquid and organic solvent had a significant influence on the enzyme-catalyzed biotransformation of (*R*,*S*)-clopidogrel carboxylic acid. This was proven by obtaining various values of conversion (c = 18.84% ± 0.68%–49.60% ± 0.57%), enantiomeric excesses of products (ee_p_ = 80.02% ± 1.57%–94.21% ± 1.07%), and enantioselectivity (E = 12.63 ± 0.14–113.40 ± 1.29). 

According to the choice of organic solvent, various reaction media were tested during the chemical esterification of clopidogrel carboxylic acid, such as acetonitrile, chloroform, cyclohexane, dichloroethane, dichloromethane, *n*-hexane, *n*-heptane, methanol, *t*-butyl-methyl ether, methyl octane, and ethyl octane. The chemical esterifications were performed using the above-mentioned organic solvents, sulfuric acid as catalysts, and methanol as the acyl acceptor. All reactions were carried out for 24 h at 37 °C. After that, all mixtures were tested in terms of the efficiency of esterification using an HPLC analysis. Based on the obtained results, cyclohexane, *n*-heptane, and *n*-hexane were selected as the most appropriate organic solvents for further experiments related to the kinetic resolution of racemic clopidogrel carboxylic acid.

Taking into account the addition of ionic liquids, it should be noted that [EMIM] [BF_4_] was the best for the enantioselective esterification of (*R*,*S*)-clopidogrel carboxylic acid among all the tested ILs in every catalytic system, as is shown in Table 1. The catalytic efficiency of *Candida rugosa* lipase varies depending on different anions of the ILs in the following order: [BF_4_]^−^ > [MSF_3_]^−^ > [MeSO_4_]^−^ > [EtSO_4_]^−^, and on different cations of the ILs as follows: [EMIM]^+^ > [HMIM]^+^ > [DMIM]^+^. As previously described in the literature, various ILs, which contain different anions and cations, could interact with the enantioselective biocatalyst in various ways, such as through ionic and dipolar interactions, hydrogen bonding, or van der Waals forces. It was previously stated that anions with a lower hydrogen bond basicity have been demonstrated as enzyme-compatible, since they do not affect the internal conformation of the enzyme’s structure [37].

**Figure 4:**
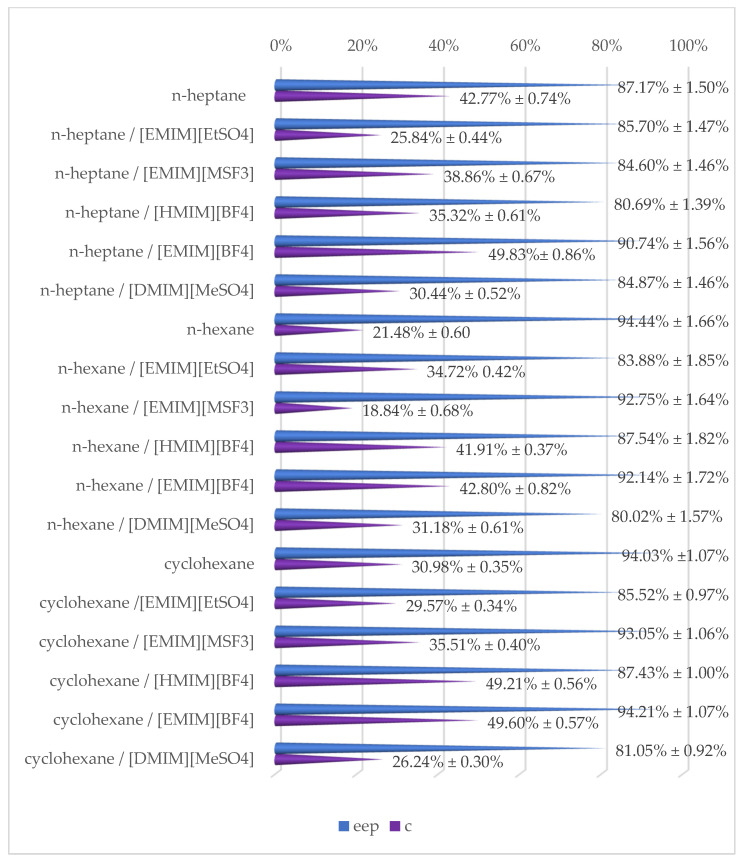
Overview of obtained results of performed enantioselective biotransformation of (*R*,*S*)-clopidogrel carboxylic acid after 120 h of incubation, including enantiomeric excesses of products (*ee_p_*) and conversion (*c*).

**Figure 5:**
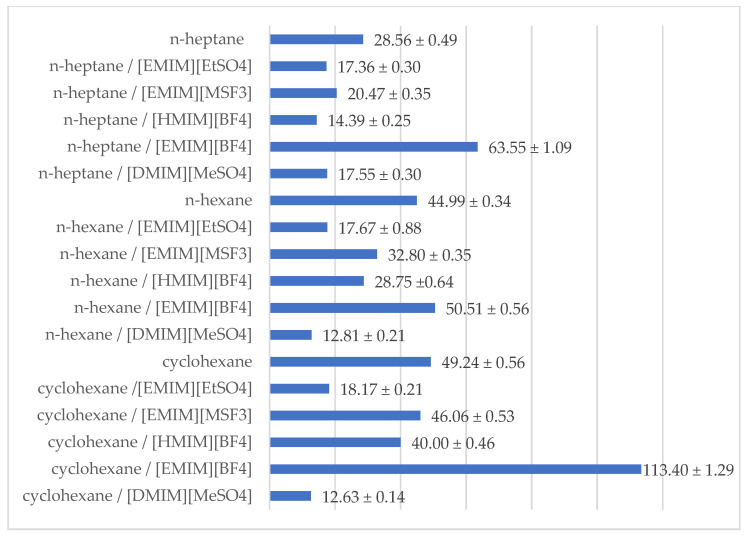
Overview of obtained results of performed enantioselective biotransformation of (*R*,*S*)-clopidogrel carboxylic acid after 120 h of incubation, including enantioselectivity (*E*).

Racemic clopidogrel carboxylic acid was fully dissolved in [EMIM][EtSO_4_], [EMIM][MSF_3_], [HMIM][BF_4_], [EMIM][BF_4_], or [DMIM][MeSO_4_], and then one of the organic solvents was added. The best kinetic resolution parameters were observed for the system containing [EMIM][BF4] and cyclohexane. In all the tested reaction systems, the enantiomeric excesses of products were higher than 80% (Figure 4). Based on the previously described studies, biotransformation could be considered enantioselective if the E-ratio is higher than 20. In Figure 5, the E-ratios of the tested systems are summed up. Each system without the addition of ionic liquids could be considered enantioselective. Nevertheless, the addition of an ionic liquid could increase the enantioselectivity of the kinetic resolution of the clopidogrel carboxylic acid. 

## 3. Materials and Methods

### 3.1. Chemicals

Acetonitrile, methanol, formic acid, [EMIM][BF_4_], [EMIM][EtSO_4_], [HMIM][BF_4_], [EMIM][MSF_3_], [DMIMMeSO_4_], *n*-heptane, *n*-hexane, and cyclohexane were purchased from Merck, Sigma-Aldrich Co., Steinheim, Germany.

(*R*,*S*)-clopidogrel carboxylic acid, (*R*,*S*)-clopidogrel, (*R*)-clopidogrel, and (*S*)-clopidogrel were purchased from Toronto Research Chemicals, Toronto, Canada.

Lipase from *Candida rugosa* OF was a gift from Meito Sangyo Co., Tachikawa, Japan. The activity of lipase from *Candida rugosa* OF was a 360,000 U/g powder. The thermal stability of lipase was equal to or below 37 °C, and the optimal pH was 6–7.

In the conducted study, the water used was obtained using a Milli-Q Water Purification System, Millipore, Bedford, MA, USA. 

### 3.2. Instrumentation

The HPLC samples were washed using refrigerated CentriVap concentrators purchased from Labconco, Kansas City, MO, USA. The Shimadzu, Kyoto, Japan, UPLC-MS/MS system used for the HPLC studies consisted of an autosampler (SIL-40AC), two solvent feed pumps with a gradient system (LC-40AD), a degasser (DGU- 30A5), a column oven (CTO-40AC), a UV detector (SPD-M20A), and a triple quadrupole mass spectrometer detector (model: LCMS-8045). 

A model KJO-4282 Guard Cartridge System and a Lux Cellulose-2 (LC-2) chiral column with a cellulose tris(3-chloro-4-methylphenylcarbamate) stationary phase, as well as a Lux Cellulose-3 (LC-3) chiral column with a cellulose tris(4-methyl benzoate) from Phenomenex Co., Torrance, CA, USA, were used in the chiral chromatographic separations. 

All incubations were performed in a dedicated incubator, model Incubators 1000 and Unimax 1010. The incubators were purchased from Heidolph, Schwabach, Germany with controlled temperature and rotation (250 RPM). Each piece of glass used was dried in an oven overnight before being cooled with a stream of nitrogen. 

### 3.3. Chromatographic Conditions

In order to optimize the chiral separations of all reagents, e.g., the enantiomers of racemic clopidogrel carboxylic acid and the enantiomers of racemic clopidogrel, various chromatographic conditions were investigated. Finally, the baseline chiral separations of the enantiomers of both clopidogrel carboxylic acid and clopidogrel were accomplished using two different chiral columns: Lux Cellulose-2 for (*R*,*S*)-clopidogrel carboxylic acid and Lux Cellulose-3 for (*R*,*S*)-clopidogrel. The composition of the mobile phase for the chiral separation of (*R*,*S*)-clopidogrel carboxylic acid included acetonitrile, methanol, and formic acid in a volumetric ratio of 87.5/12.5/0.1, whereas for the chiral separation of (*R*,*S*)-clopidogrel, the mobile phase was composed of methanol and formic acid in a volumetric ratio of 100/0.1. 

**Figure 6:**
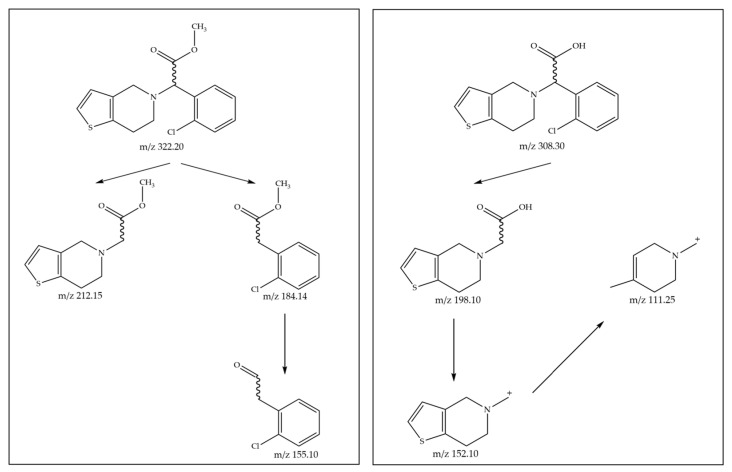
Fragmentation pathways for molecular ions of (*R*,*S*)-clopidogrel (*m*/*z* 322.20) and (*R*,*S*)-clopidogrel carboxylic acid (*m*/*z* 308.30).

In both the optimized chromatographic conditions, the mobile phase flow rate was set at 0.8 mL/min in order to obtain a proper resolution. A triple quadrupole mass spectrometer in multiple reaction monitoring modes (MRMs) was utilized to detect chiral compounds. (*R*,*S*)-clopidogrel carboxylic acid had MRM transitions of 308.30 > 198.10, 308.30 > 152.10, and 308.30 > 111.25, whereas (*R*,*S*)-clopidogrel had transitions of 322.20 > 212.15, 322.20 > 185.15, and 322.20 > 155.10 (Figure 6).

**Figure 7:**
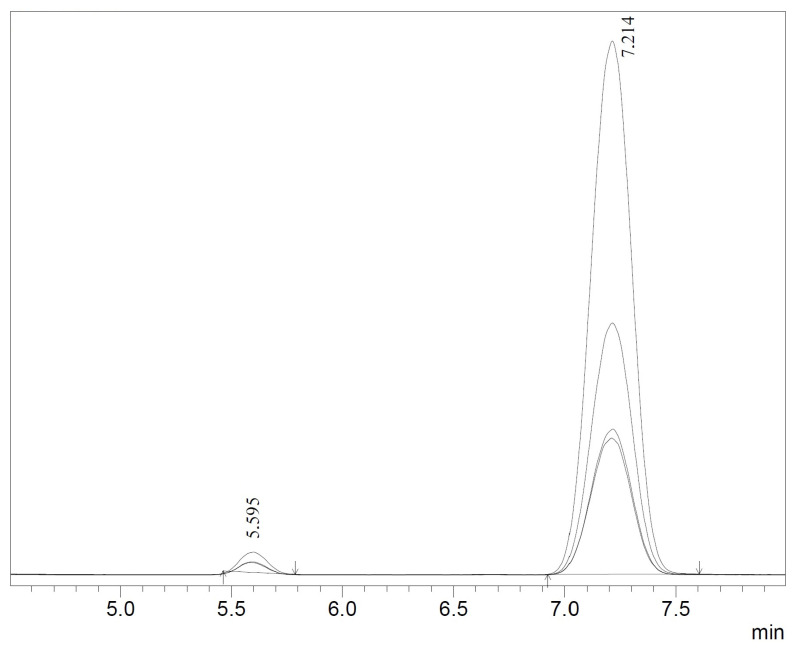
Chromatogram of (*R*,*S*)-clopidogrel after 120 h of kinetic resolution of (*R*,*S*)-clopidogrel carboxylic acid with the use of *Candida rugosa* OF in two-phase reaction media containing [EMIM] [BF_4_] and cyclohexane: (*R*)-clopidgorel (t_R_ = 5.595 min) and (*S*)-clopidogrel (t_R_ = 7.214 min). Chromatographic conditions: Lux Cellulose-3 (4.6 × 250 mm × 3 μm) column, mobile phase: methanol/formic acid (100/0.1 v/v), F_R_ = 0.8 mL/min, t = 20 °C.

The retention time of (*R*)-clopidogrel carboxylic acid was t_R_ = 5.821 min and (*S*)-clopidogrel carboxylic acid was t_R_ = 7.539 min, whereas (*R*)-clopidogrel was t_R_ = 5.595 min and (*S*)-clopidogrel was t_R_ = 7.214 min (Figure 7).

Using the equations described by Chen et al. [38] and Chen et al. [39] based on the peak areas from chromatograms of the enantiomers of clopidogrel carboxylic acid and clopidogrel, it was possible to determine the conversion and optical purity of both substrates and products, as well as the enantioselectivity of the enzyme-catalyzed biotransformation that was carried out.

### 3.4. Kinetic Resolution of (R,S)-Clopidogrel Carboxylic Acid

In a 10 mL glass flask, the enantioselective biotransformation of racemic clopidogrel carboxylic acid was performed. The reaction mixture contained (*R*,*S*)-clopidogrel carboxylic acid (3.0 mg, 0.01 mM) dissolved in 250 µL of the chosen ionic liquid and placed in 5 mL of *n*-hexane, *n*-heptane, or cyclohexane, which, when combined, constituted a two-phase reaction medium. In the samples without ionic liquids, (*R*,*S*)-clopidogrel carboxylic acid (3.0 mg, 0.01 mM) was directly dissolved in 5 mL of one of the above-mentioned organic solvents. The reaction used methanol (3 µL, 0.25 mM) as an acyl acceptor. The effect of the addition of the ionic liquids [EMIM][BF_4_], [EMIM][EtSO_4_], [HMIM][BF_4_], [EMIM][MSF_3_], or [DMIMMeSO_4_] on the kinetic resolution of (*R*,*S*)-clopidogrel carboxylic acid was a part of the investigation. By directly adding 10 mg of native lipase from *Candida rugosa* OF to the previously assembled bioreactor, the enzyme-catalyzed biotransformation of (*R*,*S*)-carboxylic acid was initiated. The reaction mixtures were incubated and shaken (250 RPM) at 37 °C.

By using a chiral stationary phase and a UPLC system coupled with a triple quadrupole mass spectrometer in MRM mode, the enantioselective biotransformation of (*R*,*S*)-clopidogrel carboxylic acid was monitored. Samples of 10 μL of ionic liquid containing the substrates and products were collected at predetermined time points every 24 h for 120 h. Next, the chiral compounds were extracted from the ionic liquid via liquid–liquid extraction by vigorous shaking with 500 μL of acetonitrile for 10 min. After centrifugation and filtration using syringe filters, the prepared samples were transfered into the vials and directly injected into a UPLC chiral column. 

## 4. Conclusions

Several other approaches previously described elsewhere allow for the obtaining of (*S*)-clopidogrel [16,40,41]. Nevertheless, more attempts to find green and economical synthetic methods recommended by the ESC P2Y12 inhibitor are still necessary. The presented study aimed to optimize the most suitable conditions to obtain enantiomerically pure (*S*)-clopidogrel at a laboratory scale. The experiment’s results support the hypothesis that *Candida rugosa* lipase OF is suitable for catalyzing the kinetic resolution of racemic clopidogrel carboxylic acid. It was proven that using the two-phase catalytic system with cyclohexane, [EMIM][BF_4_], and *Candida rugosa* lipase allowed for the obtaining of highly enantioselective parameters. The enantioselective biotransformation of racemic clopidogrel carboxylic acid was performed under various reaction conditions. The study verified the possibility of performing the kinetic resolution of racemic clopidogrel carboxylic acid in a two-phase catalytic system using methanol as an acyl acceptor. According to the available literature, it was decided to test five various ionic liquids: [EMIM][EtSO_4_], [EMIM][MSF_3_], [HMIM][BF_4_], [EMIM][BF_4_], and [DMIM][MeSO_4_] [42,43,44,45,46,47]. The used ionic liquids, however, displayed a variety of kinetic characteristics, leading to varying enantioselectivities and enantiomeric excesses of substrates and products. The catalytic efficiency of the *Candida rugosa* lipase varied depending on the different anions of the ILs in the following order: [BF_4_]^−^ > [MSF_3_]^−^ > [MeSO_4_]^−^ > [EtSO_4_]^−^, and on the different cations of the ILs as follows: [EMIM]^+^ > [HMIM]^+^ > [DMIM]^+^. As has been previously described in the literature, various ILs, which contain different anions and cations, could possibly interact with the enantioselective biocatalyst in various ways, such as through ionic and dipolar interactions, hydrogen bonding, or van der Waals forces [37]. Nevertheless, it is important not to affect the enzyme conformation. Therefore, it seems that factors such as the reaction medium influence the enzyme conformation and could decrease or increase the enantioselective properties of biocatalysts. Although the biotransformations were performed at a laboratory scale, the optimized conditions and the described observations could be considered significant milestones for further scaling up the production and for possible industrial synthesis, which is planned.

It should be stressed that two-phase catalytic systems containing ionic liquids can be critical from an economic point of view, as they allow for a direct and substantial reduction in the overall cost of biotransformations catalyzed by enzymes. Since the biocatalysts can be easily separated from the substrate and the product, the lipase can be reused in another reaction. This approach has been comprehensively described elsewhere in kinetic resolutions of various racemic compounds using ionic liquids [29,48,49]. Furthermore, it should be emphasized that the solubility of racemic clopidogrel carboxylic acid in organic solvents is relatively low and requires more significant volumes of the reaction medium, which could have a negative impact on the environment. Therefore, the future prospects of the presented study should be referred to when seeking a new functionality for reaction mixtures, which, apart from obtaining better and more efficient reaction parameters, should also be “greener” and re-usable. 

## Figures and Tables

**Table 1:** List of obtained results of performed enantioselective esterification of (*R*,*S*)-clopidogrel carboxylic acid after 5 days (120 h) of reaction: enantiomeric excesses of substrates (*ee_s_*), products (*ee_p_*), conversion (*c*), and enantioselectivity (*E*).

Reaction Medium		ee_p_	ee_s_	c	E
Organic Solvent	Ionic Liquid				
*n*-heptane	-	87.17% ±1.50%	65.15% ±1.12%	42.77% ±0.74%	28.56 ±0.49
*n*-heptane	[EMIM][EtSO_4_]	85.70% ±1.47%	29.85% ±0.51%	25.84% ±0.44%	17.36 ±0.30
*n*-heptane	[EMIM][MSF_3_]	84.60% ±1.46%	53.77% ±0.92%	38.86% ±0.67%	20.47 ±0.35
*n*-heptane	[HMIM][BF_4_]	80.69% ±1.39%	44.06% ±0.76%	35.32% ±0.61%	14.39 ±0.25
*n*-heptane	[EMIM][BF_4_]	90.74% ±1.56%	90.11% ±1.55%	49.83% ±0.86%	63.55 ±1.09
*n*-heptane	[DMIM][MeSO_4_]	84.87% ±1.46%	37.15% ±0.64%	30.44% ±0.52%	17.55 ±0.30
*n*-hexane	-	94.44% ±1.66%	25.83% ±0.73%	21.48% ±0.60%	44.99 ±0.34
*n*-hexane	[EMIM][EtSO_4_]	83.88% ±1.85%	44.61% ±0.51%	34.72% ±0.42%	17.67 ±0.88
*n*-hexane	[EMIM][MSF_3_]	92.75% ±1.64%	21.53% ±0.87%	18.84% ±0.68%	32.80 ±0.35
*n*-hexane	[HMIM][BF_4_]	87.54% ±1.82%	63.16% ±0.42%	41.91% ±0.37%	28.75 ±0.64
*n*-hexane	[EMIM][BF_4_]	92.14% ±1.72%	68.95% ±1.24%	42.80% ±0.82%	50.51 ±0.56
*n*-hexane	[DMIM][MeSO_4_]	80.02% ±1.57%	36.25% ±0.71%	31.18% ±0.61%	12.81 ±0.25
cyclohexane	-	94.03% ±1.07%	42.20% ±0.48%	30.98% ±0.35%	49.24 ±0.56
cyclohexane	[EMIM][EtSO_4_]	85.52% ±0.97%	35.90% ±0.41%	29.57% ±0.34%	18.17 ±0.21
cyclohexane	[EMIM][MSF_3_]	93.05% ±1.06%	50.78% ±0.58%	35.31% ±0.40%	46.06 ±0.53
cyclohexane	[HMIM][BF_4_]	87.43% ±1.00%	84.70% ±0.97%	49.21% ±0.56%	40.00 ±0.46
cyclohexane	[EMIM][BF_4_]	94.21% ±1.07%	92.71% ±1.06%	49.60% ±0.57%	113.40 ±1.29
cyclohexane	[DMIM][MeSO_4_]	81.05% ±0.92%	28.83% ±0.33%	26.24% ±0.30%	12.63 ±0.14

*ee_p_*—enantiomeric excesses of products; *ee_s_*—enantiomeric excesses of substrates; *c*—conversion; *E*—enantioselectivity.

## Data Availability

Not applicable.
